# GLP-1 receptor agonists: a magic bullet for obesity?

**DOI:** 10.1016/j.lanwpc.2024.101233

**Published:** 2024-10-31

**Authors:** The Lancet Regional Health – Western Pacific

For the first time in decades, the prevalence of obesity has not increased in the USA, according to the latest National Health and Nutrition Examination Survey from the US Centers for Disease Control and Prevention in September, 2024. Both obesity and severe obesity prevalences are similar between 2021–23 and 2017–20. The reasons behind the changing trend were not discussed, but it is worth noting that the time frame concurs with the introduction of GLP-1 receptor agonists (GLP-1RAs) for weight management in the country.

GLP-1RAs were originally developed for the treatment of type 2 diabetes. The US Food and Drug Administration and other health authorities have since approved the use of GLP-1RAs, such as semaglutide and liraglutide, for weight management, owing to their effect on weight reduction in clinical trials. In addition to glucose control and weight loss, these medicines have shown beneficial effects in reducing overweight and obesity related cardiovascular risk and improving kidney outcomes. It is encouraging to see the positive impact of these medicines on weight reduction across different populations. The recent STEP 7 trial, along with the STEP 6 trial published in 2022, indicates that semaglutide (2.4 mg) has similar effects on weight reduction in eastern Asian populations. Specifically, participants with overweight or obesity experienced an average weight reduction of 12.1% over a period of 44 weeks in the STEP 7 trial. These data are welcomed by the Asia–Pacific region where countries are experiencing an alarming rate of increase in obesity.

The use of GLP-1RAs is not without side-effects, such as gastrointestinal disorders when used at higher doses, and bodyweight regain after discontinuing the medication. A broader application of these medicines as public strategies for obesity treatment needs to be approached with caution, considering cost and accessibility. The current price for GLP-1RAs for weight management is high for both individuals and the health-care system, with costs varying across different countries. For example, a 4-week supply costs US $1349.02 in the USA and $283.12 in Japan. Insurance coverage for GLP-1RAs for weight management is only included in a few high-income countries for selected populations with higher BMIs and other comorbidities. Cost and cost-effectiveness are the main concerns for many countries that do not reimburse GLP-1RAs with public funds. As a result, people often turn to unofficial sources to purchase the medication, creating an allocation system based on the ability to pay. This inequitable access to GLP-1RAs, along with a global drug shortage since 2022, reaffirms the urgent need for effective obesity treatment options. Achieving this requires more evidence on real-world costs, accessibility, and the long-term safety profile of GLP-1RAs, especially in the Western Pacific region, where such data are currently limited.

The discussion surrounding the cost, accessibility, and safety of GLP-1RAs is not just about introducing novel pharmacological therapies, but also offers an opportunity to reconsider our attitude towards obesity and its management. While obesity is increasingly recognised as a chronic health condition, it is still often perceived as a consequence of personal choices related to unhealthy lifestyles and a lack of willingness to manage weight. A study conducted in south and southeast Asian countries revealed that people with obesity tend to attribute the failure of weight control to personal reasons, such as not following dietary and exercise guidelines or losing motivation. This oversimplified attribution of obesity leads to and amplifies weight stigma, which can hamper patients from seeking medical advice, discourage clinicians from recognising and delivering effective treatment options, and limit incentive and motivation to prioritise obesity management in policy making. When public discussions concentrate on the price of GLP-1RAs, it should not be ignored that many costly treatment options are covered for diseases like certain cancers and cardiovascular diseases, which are knowingly associated with unhealthy lifestyles and personal decisions.

Obesity is a complex health condition associated with multiple genetic, socioeconomic, and environmental determinants, which could be prevented at the very beginning. GLP-1RAs “are reshaping not only how obesity is treated, but how it's understood—as a chronic illness with roots in biology, not a simple failure of willpower”, mentioned in the 2023 Science Breakthrough of the Year. Holistic approaches to reducing stigma, enhancing public education, and applying possible treatment options—including lifestyle intervention, pharmacological therapy, and weight loss surgery—are essential to create a supportive environment for weight management and improving overall public health outcomes.

